# Supplementary material for: A Comparison of the Costs and Benefits of Bacterial Gene Expression
Source: PLoS One. 2016 Oct 6;11(10):e0164314. doi: 10.1371/journal.pone.0164314 (PMC5053530; doi:10.1371/journal.pone.0164314)
Supplement: S1 Fig — (PDF) [file pone.0164314.s001.pdf]

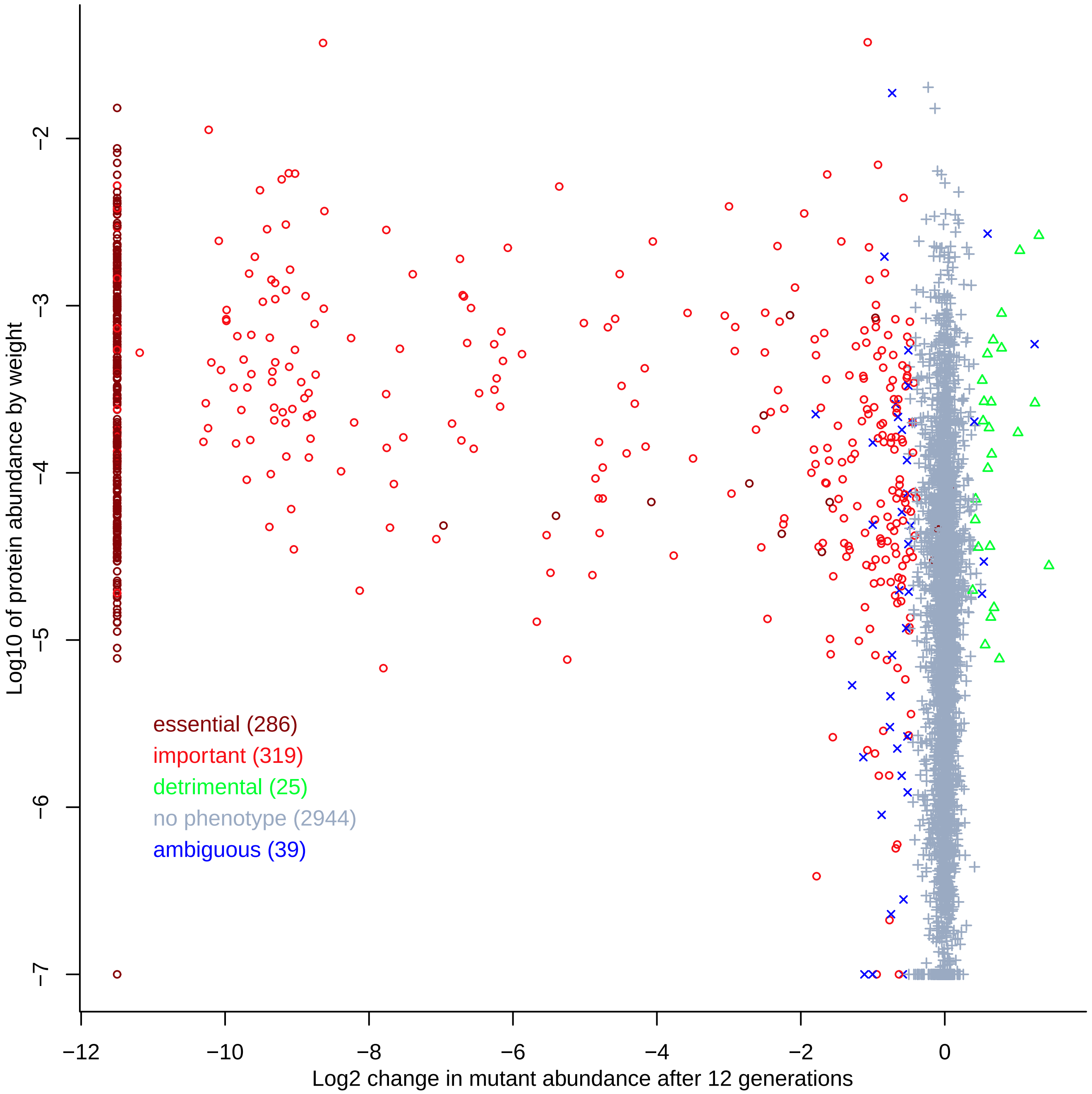

Supplementary Figure S1: Scatter plot of gene fitness versus protein expression. For each *E. coli* protein, we show the $\log_{10}$ protein expression (by weight) versus the $\log_2$ change in mutant abundance after 12 generations of growth in glucose minimal media. Proteins whose expression was not detected by ribosomal profiling are shown at the bottom (an abundance of 10^{-7}). Mutants in many of the essential proteins (from PEC), as well as mutants in some important proteins (as identified by growth curves of the Keio collection), had inadequate abundance at the beginning of our experiment, so that we could not estimate their change in mutant abundance. They probably cannot grow in our media and are shown at the left (a $\log_2$ change of -11.5). The plot includes 39 ambiguous proteins that had noticeable but non-significant impacts on fitness, but another 449 ambiguous proteins are not shown because of insufficient coverage to estimate gene fitness.
