# Supplementary material for: A Comparison of the Costs and Benefits of Bacterial Gene Expression
Source: PLoS One. 2016 Oct 6;11(10):e0164314. doi: 10.1371/journal.pone.0164314 (PMC5053530; doi:10.1371/journal.pone.0164314)
Supplement: S2 Fig — (PDF) [file pone.0164314.s002.pdf]

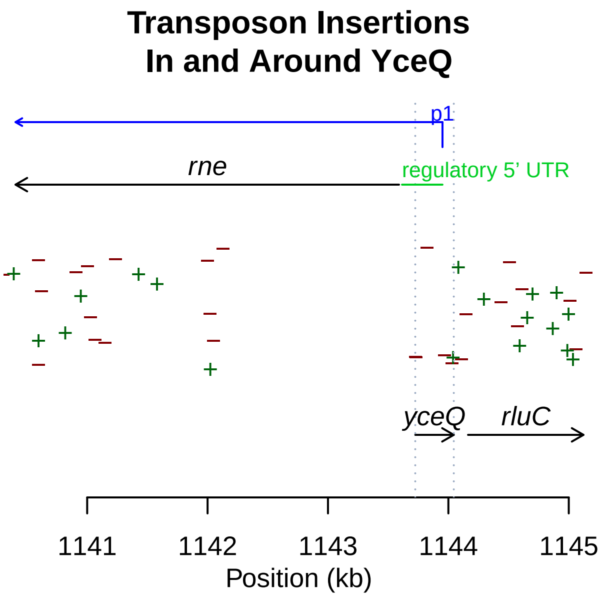


**Supplementary Figure S2: Locations of transposon insertions in and around yceQ.** Only locations that are supported by at least two TnSeq reads (Wetmore et al 2015) are shown. A "+" symbol indicates that the promoter and the antibiotic resistance gene within the transposon are on the + strand. We also highlight the location of the primary promoter (p1) of *rne* (Ow et al, Molecular microbiology 43:159-171), which is an essential gene, and the conserved and structured regulatory leader in the 5' untranslated region of the *rne* mRNA (Diwa et al, Genes & development 14:1249-60). The vertical lines show the extent of *yceQ*. Note that *yceQ* overlaps both the primary *rne* promoter and the regulatory leader, which may explain why deleting the entire region is not possible (Baba et al 2006). Consistent with this, we identified transposon insertions within *yceQ* but only in the "-" orientation, so that the promoter within the transposon could drive expression of *rne*. Incidentally, insertions within the 3' part of *rne* are viable because the C-terminal part of RNase E is not required for its catalytic activity or for its essential role in processing ribosomal RNA (Kido et al, J. Bacteriology 187:3917-25; Lopez et al, Molecular microbiology 33:188-99).
