# Supplementary material for: A Comparison of the Costs and Benefits of Bacterial Gene Expression
Source: PLoS One. 2016 Oct 6;11(10):e0164314. doi: 10.1371/journal.pone.0164314 (PMC5053530; doi:10.1371/journal.pone.0164314)
Supplement: S3 Fig — (PDF) [file pone.0164314.s003.pdf]

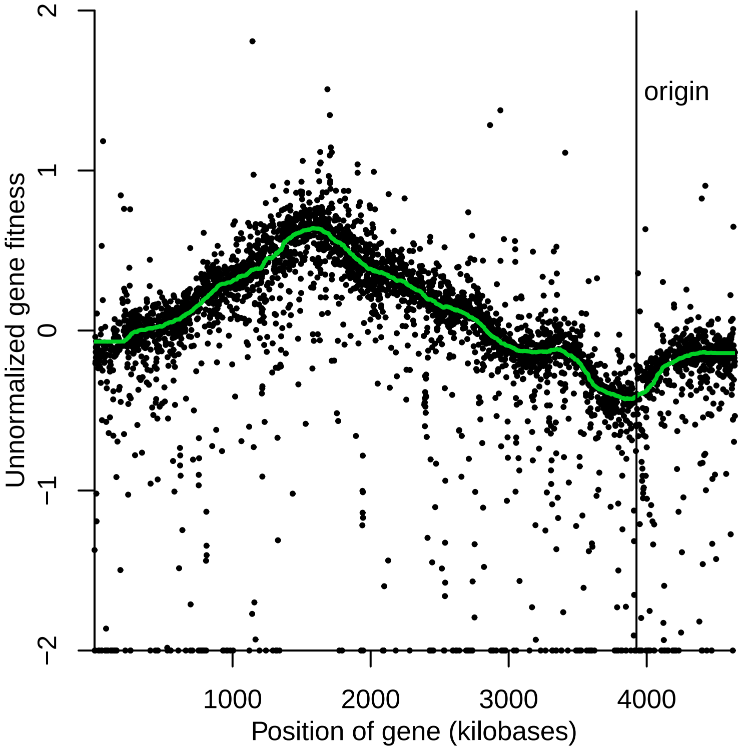

Supplementary Figure S3: Normalization of gene fitness values by location on the chromosome. The points show the unnormalized gene fitness values after 12 generations versus the position along the chromosome. The line shows an estimate of the bias due to chromosomal position (the running median). We also highlight the location of the origin of replication. The fitness values (and the estimated bias) are the average of 2 replicates, and fitness values below -2 are shown at -2. The bias is the lowest at the origin because the control sample was growing in rich media, so it was growing faster than the experimental samples. Conversely, the bias is the highest around the terminus. Hence, the bias should be a “V” shape with the minimum at the origin and the maximum at the terminus, as observed.
